# EHMTI-0052. Efficacy of early vs. late use of frovatriptan combined with dexketoprofen vs. frovatriptan alone in the acute treatment of migraine attacks with or without aura

**DOI:** 10.1186/1129-2377-15-S1-G3

**Published:** 2014-09-18

**Authors:** G Allais, V Tullo, P Cortelli, P Barbanti, F Valguarnera, G Sette, F D'Onofrio, M Curone, G Reggiardo, S Omboni, F Frediani, G Bussone, C Benedetto

**Affiliations:** 1 Department of Gynecology and Obstetrics, Women's Headache Center, Torino, Italy; 2 Clinical Neuroscience, National Neurological Institute Carlo Besta, Milano, Italy; 3 Neurological Science, University of Bologna, Bologna, Italy; 4 Headache and Pain Unit Department of Neurological Motor and Sensorial Sciences, IRCCS San Raffaele Pisana, Roma, Italy; 5 Department Head-Neck, Sestri Ponente Hospital, Genova, Italy; 6 Neurological Headache Centre, Sant'Andrea Hospital, Roma, Italy; 7 Neurologic Unit, San Giuseppe Moscati Hospital, Avellino, Italy; 8 Biometric Unit, Mediservice, Milano, Italy; 9 Clinical Research Unit, Italian Institute of Telemedicine, Solbiate Arno (Varese), Italy; 10 Headache Center, Ospedale San Carlo Borromeo, Milano, Italy

## Aims

To compare frovatriptan 2.5 mg plus dexketoprofen 25 or 37.5 mg (FroDex25 and FroDex37.5) vs. frovatriptan 2.5 mg (Frova) in the acute treatment of migraine attacks in a post-hoc analysis of a double-blind, randomized, parallel-group study. Patients who took the drug within 30 min from the onset of pain (early use, EU) or after (late use, LU) were analyzed.

## Methods

314 migraineurs with or without aura treated at least one attack with Frova, FroDex25 or FroDex37.5. Traditional migraine endpoints were compared across study drugs in the 279 patients of the full analysis set according to EU (n=172) or LU (n=107).

## Results

Pain free (PF) at 2-hours in EU was 33% with Frova, 50% with FroDex25 and 51% with FroDex37.5 (p=NS), while in LU was 22%, 51% and 50% (p<0.05 combinations vs. monotherapy), respectively. PF at 4-hours was 54% for EU and 34% for LU of Frova, 71% and 57% with FroDex25 and 74% and 68% with FroDex37.5 (p<0.05 for EU and p<0.01 for LU vs. Frova). Sustained pain free at 24-hours was 26% under Frova, 43% under FroDex25 and 40% under FroDex37.5 (p=NS) in EU, while it was 19% under Frova, 43% under FroDex25 and 45% under FroDex37.5 (p<0.05 FroDex25 and FroDex37.5 vs. Frova) in LU. Risk of relapse at 48-hours was similar (p=NS) among study groups (Frova: 25%, FroDex25: 21%, and FroDex37.5: 37%) for both EU and LU (14%, 42% and 32%).

## Conclusions

FroDex was found to be more effective than Frova taken either early or late.

